# Assessment of the Diagnostic Efficacy of Low-Field Magnetic Resonance Imaging: A Systematic Review

**DOI:** 10.3390/diagnostics14141564

**Published:** 2024-07-19

**Authors:** Barbora Mašková, Martin Rožánek, Ondřej Gajdoš, Evgeniia Karnoub, Vojtěch Kamenský, Gleb Donin

**Affiliations:** Department of Biomedical Technology, Czech Technical University in Prague, 272 01 Kladno, Czech Republic; barbora.klicova@fbmi.cvut.cz (B.M.); gajdoond@fbmi.cvut.cz (O.G.); evgeniia.mardanshina@fbmi.cvut.cz (E.K.); kamenvoj@fbmi.cvut.cz (V.K.); gleb.donin@fbmi.cvut.cz (G.D.)

**Keywords:** low field magnetic resonance, magnetic resonance, diagnostic efficacy, diagnostic accuracy

## Abstract

Background: In recent years, there has been an increasing effort to take advantage of the potential use of low magnetic induction devices with less than 1 T, referred to as Low-Field MRI (LF MRI). LF MRI systems were used, especially in the early days of magnetic resonance technology. Over time, magnetic induction values of 1.5 and 3 T have become the standard for clinical devices, mainly because LF MRI systems were suffering from significantly lower quality of the images, e.g., signal–noise ratio. In recent years, due to advances in image processing with artificial intelligence, there has been an increasing effort to take advantage of the potential use of LF MRI with induction of less than 1 T. This overview article focuses on the analysis of the evidence concerning the diagnostic efficacy of modern LF MRI systems and the clinical comparison of LF MRI with 1.5 T systems in imaging the nervous system, musculoskeletal system, and organs of the chest, abdomen, and pelvis. Methodology: A systematic literature review of MEDLINE, PubMed, Scopus, Web of Science, and CENTRAL databases for the period 2018–2023 was performed according to the recommended PRISMA protocol. Data were analysed to identify studies comparing the accuracy, reliability and diagnostic performance of LF MRI technology compared to available 1.5 T MRI. RESULTS: A total of 1275 publications were retrieved from the selected databases. Only two articles meeting all predefined inclusion criteria were selected for detailed assessment. Conclusions: A limited number of robust studies on the accuracy and diagnostic performance of LF MRI compared with 1.5 T MRI was available. The current evidence is not sufficient to draw any definitive insights. More scientific research is needed to make informed conclusions regarding the effectiveness of LF MRI technology.

## 1. Introduction

The first magnetic resonance imaging (MRI) systems developed for clinical use (around 1980) had a magnetic field intensity of less than 0.35 T [1]. With the gradual development of MRI systems, significant advances have been made, one of which was the improvement in static magnetic field, with conventional 1.5 T and 3 T MRI systems becoming conventional systems used for clinical applications, replacing Low-Field MRI (LF MRI) [2]. High-field systems have gained a dominant market share due to higher resolution, higher SNR (Signal to Noise Ratio) per unit of time, greater contrast sensitivity, and more advanced sequence [1]. This review focuses on LF MRI, which is defined as a system with a magnetic field strength below 1 Tesla [3].

Although commercial lower-field devices were available, many were rarely used or have been discontinued altogether. However, commercial interest (in 2018) has led to the FDA (Food and Drug Administration) approval of several low-field systems. Hyperfine was the first to market its device, the MRI 0.064 T Hyperfine Swoop (head examination), followed by the MRI 0.066 T Promaxo (prostate examination), the MRI 0.5 T Synaptive Evry (intraoperative MRI), and the MRI 0.55 T Siemens Magnetom Free.Max (whole-body) [3,4]. While high-field devices have gained market dominance based on the parameters already mentioned, there are two main factors that create opportunities for current low-field systems. One factor is the lower acquisition costs, and the other is technological innovations that lead to improved image quality [5].

Due to the development in MRI device hardware and software, including, for example, new coils or rather innovative pulse sequences and advanced processing of measured signals (using Deep Learning and Denoising Techniques), it is possible to reduce noise in the image by methods other than increasing magnetic induction and to improve the overall quality of the resulting image [1,5].

The aim of this article is to assess the diagnostic efficacy and capability of clinically available LF MRI technology in the obtaining of clinically relevant results in comparison with 1.5 T MRI devices in imaging the nervous system (brain, spinal cord, spine), musculoskeletal system (joints, bones, muscles) and organs of the chest, abdomen, and pelvis. To achieve this goal, studies comparing these technologies according to their diagnostic outcomes, their impact on decision-making in clinical settings, and their subsequent impact on the treatment process and its clinical outcomes were analysed.

The manuscript provides current evidence of the diagnostic performance of LF MRI, although it is limited. The findings may offer valuable insights for clinical practice and contribute to a better understanding of the appropriateness and effectiveness of LF MRI in diagnostic practice.

The manuscript has the following structure. Elementary steps of systematic review are described in Materials and Methods. The list of studies that were completely read and evaluated are summarised in Results. Limitations and perspectives are discussed in the Discussion.

## 2. Materials and Methods

The analysis of studies was carried out in the form of a systematic review; the recommended Preferred Reporting Items for Systematic Reviews and Meta-Analyses (PRISMA) procedure was used for this analysis [6]. The study is not registered in PROSPERO. Within the specific methods and procedures, the recommendations of the Cochrane Collaboration were also taken into account [7]. Publications from 2018 to January 2023 were included in the analysis, the reason being the novelty of the technology. Several LF MRIs were cleared by the FDA in 2018 [8].

For conducting the systematic review, a key question was established: What evidence exists regarding the diagnostic efficacy of clinically available whole-body scanners with low magnetic inductance compared to 1.5 T MRI devices in imaging the nervous system (brain, spinal cord, spine), musculoskeletal system (joints, bones, muscles), and organs of the chest, abdomen, and pelvis?

The diagnostic efficacy of imaging technologies is a complex issue, and it is influenced by many factors. The hierarchical model proposed by Fryback and Thornbury is often used to assess it [9]. This model describes the individual steps that should be considered when assessing the clinical value of diagnostic imaging methods. According to this model, the diagnostic efficacy of an imaging technology can be viewed at several levels:

- Level 1: technical quality of the test;
- Level 2: diagnostic accuracy, sensitivity and specificity of the test, interpretation;
- Level 3: whether the test result leads to a change in the diagnostic thinking of the physician;
- Level 4: impact of the test on the patient’s treatment plan;
- Level 5: impact of the test on patient outcomes;
- Level 6: societal costs and benefits of the diagnostic test.

The analysis of the literature was primarily focused on levels 2 to 5 of the Fryback and Thornbury model, as this level is directly related to the terms of reference of the study [9].

### 2.1. Sources and Search Strategies

A systematic review was performed in the MEDLINE, PubMed, CENTRAL, Web of Science and Scopus databases to identify studies comparing the accuracy, reliability, and diagnostic efficacy of currently available LF MRI technologies. The search was conducted once at the end of 2023. Two researchers conducted independent data extraction and quality assessment. Papers in English and German language have been analysed.

Searches were conducted using standard MeSH terms as well as specific free terms and their combinations related to the key questions of the review. A detailed description of the search strategies and a list of the search terms used is provided in Table S1.

PubMed searches were limited to records that were not indexed in the MEDLINE database. Searches in the Web of Science and Scopus databases, due to their multidisciplinary nature, were limited to publications on diagnostic accuracy of tests according to the comprehensive version of the search strategy of Bachmann et al. [10].

### 2.2. Inclusion and Exclusion Criteria

The review sought to identify all primary studies comparing these technologies, namely randomised controlled trials, cohort studies, and case-control studies. Case studies, casuistries, simulation studies, etc., have not been analysed. An indirect comparison of two technologies (i.e., based on studies assessing the diagnostic characteristics of each technology without directly comparing them) would be associated with heterogeneity in estimated diagnostic accuracy across studies, and this could bias the results of the final comparison. This systematic review was based on studies that made direct comparisons between LF MRI and 1.5 T MRI technologies, either by applying both tests to each individual or by dividing subjects into groups to be tested with one technology.

The criteria for inclusion and exclusion of publications were established in advance using the objectives of this assessment and the key questions identified.

#### 2.2.1. Inclusion Criteria

All studies comparing the diagnostic performance of LF MRI technology and currently available 1.5 T MRI technologies were considered for inclusion. Publications from 2018 onwards were included in the analysis since the assessed technology is quite new. Studies were included in the subsequent analysis if

- These are clinical trials, i.e., randomised controlled trials, cohort studies or case-control studies;
- Were published in a peer-reviewed journal;
- LF MRI technology was compared with one or more currently available 1.5 T MRI technologies for one (or more) of the areas listed in the key questions;
- Technologies were compared according to their diagnostic efficacy (see Fryback and Thornbury’s model above) [9].

#### 2.2.2. Exclusion Criteria

The following types of studies or publications were excluded:

- Overview articles, reviews, casuistries, letters to the editor, commentaries, case studies;
- Studies with less than 5 patients;
- Animal, in vitro or cadaver studies;
- Abstracts from conferences that did not result in a peer-reviewed publication.

### 2.3. Screening and Assessment of Studies

Publication lists were imported into EndNote X6 (Thomson Reuters, Toronto, ON, Canada), and duplicate articles were removed. Subsequently, the remaining publications were exported to the Rayyan web-based software for screening purposes. Screening consisted of analysing the title, abstract, and other parameters of the publications. The aim of this step was to exclude obviously irrelevant publications from further analysis based on exclusion criteria.

For the records not excluded in the screening, their full texts were retrieved, and these articles proceeded to a detailed assessment of their relevance in terms of predefined inclusion and exclusion criteria. At the same time, the reference lists for these articles were manually analysed to identify any relevant studies that may have been missed in the search. The excluded publications with the reason for exclusion are listed in Supplementary Materials.

### 2.4. Data Extraction

A standardised procedure was used to obtain data from each of the included studies. The following information was obtained from the included studies: year of publication, study design, number of subjects, characteristics of the study population, imaging area, diagnostic outcomes assessed, information on the procedures used to measure them, information on test results, and other relevant information.

### 2.5. Critical Assessment

The included studies assessed the diagnostic properties of magnetic resonance imaging in organ imaging. Some studies achieved this by assessing the technical quality of the images and the reproducibility of individual measurements, while others assessed the diagnostic accuracy of the technologies. The risk of bias was assessed using the QUADAS-2 (Quality Assessment of Diagnostic Accuracy Studies 2) tool (Table S3) [11]. Two independent reviewers conducted the assessment. The search was conducted once at the end of 2023.

### 2.6. Data Analysis

The studies included in the final analysis differed significantly in their design and clinical outcomes analysed. For this reason, a formal meta-analysis of their results was not performed.

For studies aimed at comparing the results of continuous parameter measurements, it was not possible to calculate inter-rater reliability parameters (agreement among observers), inter-method reliability (agreement between the individual methods of measurement) and other key parameters.

The results of the study were used to calculate the diagnostic characteristics of the test (sensitivity and specificity), which were then recalculated in relation to the gold standard.

## 3. Results

**Figure 1:**
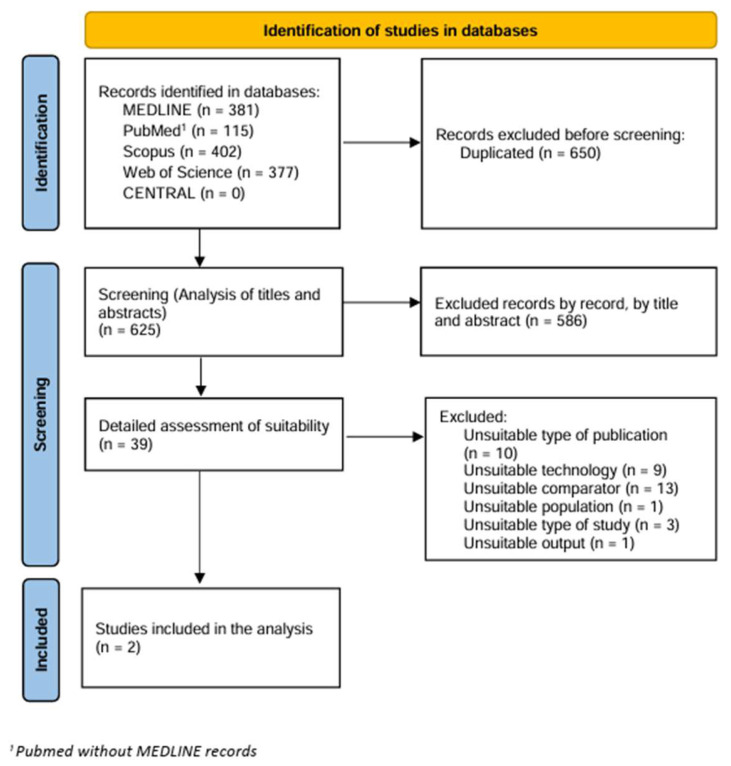
PRISMA diagram.

The export from the databases took place on 14 January 2023. The total number of results obtained from the above-defined databases was 1275 publications. From this set, 650 duplicates were removed, and the remaining 625 records were examined as part of the Phase 1 screening. From this sample, 586 non-relevant publications were removed based on titles and abstracts, and the remaining 39 proceeded to the next phase. The full texts of these 39 publications were thoroughly analysed as part of a detailed assessment of the relevance of the publications. The most common reason for exclusion from subsequent analysis was the use of an inappropriate comparator. Other reasons were inappropriate publication type or inappropriate technology. The entire selection process is illustrated by the PRISMA diagram in the following Figure 1, with the excluded studies and the reason for their exclusion listed in Supplementary Materials. Finally, 2 studies were selected that met all the predefined inclusion criteria.

### 3.1. Excluded Studies

During the assessment of suitability, a total of 37 studies were excluded (Table 1). The reasons for the exclusion of each study are detailed in Table 1. The most common reason for exclusion was inappropriate comparators, such as not comparing an LF MRI technology to a 1.5 T MRI scanner or the use of unsuitable technology, such as studies conducted on a prototype 0.55 LF MRI or MRI with induction adjustment to 0.55 T. It is worth noting that studies excluded due to inappropriate technology have served as comparative studies on so-called prototype 0.55 T MRI prior to market introduction. Comparative studies on prototypes were excluded because these prototypes represented experimental devices, were not commercially available, and had different technical parameters compared to the commercially available devices.

### 3.2. Characteristics of the Studies

The following table (Table 2) summarises the basic characteristics of the included studies [49,50]. All studies were single-centre with a small number of patients. Both studies focused on imaging of the brain region, one on imaging of intracranial aneurysms and the other on stroke diagnosis.

The study by Osmanodja et al. [49] had the main objective to assess the performance of 0.55 T MRI technology in the diagnosis of intracranial aneurysms compared with digital subtraction angiography. As part of the partial results, the authors of the study also focused on the comparison of Siemens Free.Max technology with 1.5 T MRI devices. Nine patients were included in the comparison (1.5 T vs. 0.55 T) who were suspected of having intracranial aneurysms based on previous 1.5 T MRI images. These patients subsequently underwent examination on a 0.55 T MRI machine by TOF (time-of-flight) MRI angiography. The resulting images were evaluated by two raters using Syngo.via software (Siemens Healthineers AG, Erlangen, Germany). The evaluation consisted of measuring the size of the aneurysms. The results of the measurements were averaged between raters and compared using the Wilcoxon matched-pairs test. The results of this test showed no statistically significant differences in measured aneurysm sizes between the two technologies.

This study [49] is characterised by a very small number of patients. In addition, there is a discrepancy in the description of the number of patients: at the beginning, the authors state that there were only two men in the initial cohort, but in the results, they state that there were three. It should also be noted that not all examinations on the 1.5 T MRI machine were performed at the same facility; only 3 of the 9 examinations were performed at the same hospital as the 0.55 T MRI examination. In the section dedicated to the comparison of 1.5 T and 0.55 T MRI technologies, the authors of the study did not perform a detailed analysis of the agreement among image raters or a comprehensive analysis of the agreement between the results of the measurements of the two technologies (e.g., using intraclass correlation analysis and similar approaches). At the conclusion of the study, the authors state that a further blinded study with a larger sample size is needed to confirm their results.

Rusche et al. [50] published a study in 2022 to assess the outcomes of imaging patients with strokes using a low-induction MRI device. A total of 24 patients were included in the study (14 stroke patients, 10 control patients). Patients first underwent a 1.5 T MRI scan and then a 0.55 T scan. Imaging was performed using DWI/ADC (diffusion-weighted image—apparent diffusion coefficient) and FLAIR (fluid-attenuated inversion recovery) sequences. On the images, two neuroradiologists independently and blinded evaluated the presence of the stroke, number, and localisation of lesions. The acquired images were also scored using a 10-point scale by two neuroradiologists, unblinded, in five dimensions: overall image quality, resolution, noise, contrast, and diagnostic quality. The authors did not provide a more detailed definition of each dimension. Subsequently, segmentation of lesion volumes was also performed by two additional radiologists.

The results of the image quality ratings from 17 patients using a 10-point scale were averaged among raters in each dimension and compared using the Wilcoxon test. Subsequently, inter-reader reliability was compared using the intraclass correlation coefficient (ICC). For the DWI/ADC sequence, the overall image quality, resolution, contrast, and diagnostic quality were significantly better for the 1.5 T MRI device, and the noise rating for the 0.55 T MRI device was significantly better than that of the 1.5 T MRI device. For the FLAIR sequence, overall image quality, noise and diagnostic quality were significantly better for the 1.5 T MRI technology, and no significant differences were observed between technologies for resolution and contrast. There was moderate to high agreement among raters in the results (ICC 0.64 to 0.87).

The results of stroke diagnosis were presented for 24 patients (14 in the stroke group and 10 in the control group). For the DWI/ADC sequence, rater 1 achieved similar sensitivity (92.9% [95% CI 66.1–99.8%]) and specificity (100% [95% CI 69.2–100.0%]) values for both technologies compared. Rater 2 had a lower sensitivity for the 0.55 T MRI technology (85.7% [95% CI 57.2–98.2%] vs. 100% [76.8–100.0%]) and the same specificity (100% [95% CI 69.2–100.0%]) for the compared technologies. For the FLAIR sequence, the study authors did not provide sufficiently detailed results to compare the sensitivity and specificity of the technologies in question. No significant differences were found between the 0.55 T and 1.5 T technologies in the measurement of lesion volumes.

This study [50] suffers from some methodological flaws. Patients with poor image quality for any technology were excluded from the study. The authors do not provide the criteria for assessing image quality or the number of patients excluded because of poor image quality. Furthermore, patients were excluded from the study due to a significant time difference between examinations on 0.55 T and 1.5 T devices, and patients were not excluded at the beginning of the study but only at the stage of comparing the diagnostic efficacy of the technologies (detection of stroke, etc.). In the conclusion of the study, the authors point out that a so-called convenient sample was used with limited representativeness in relation to the general population. The study also did not adequately describe the approach to selecting patients for the control group. All the above limitations of the study may result in a high risk of bias in the results of this study.

The summary results of all included studies are presented in the following Table 3.

### 3.3. Quality Assessment

The QUADAS tool was employed to assess the risk of bias (Table S3). Two studies were evaluated, wherein, in line with the research question, one study was assessed to have a high risk in the patient selection domain. The other exhibited a risk in the flow and timing domain. For instance, in the study Rusche 2022 [50], the patient selection process for the control group needed to be more adequately described. In the study Osmanodja 2023 [49], although the same reference standard (1.5 T) was utilised, not all examinations were conducted at the same facility. These are factors that could introduce bias into the outcomes.

## 4. Discussion

We found only two papers that directly compare the quality of images acquired with a full-body scanner with low magnetic induction and a conventional MRI machine using 1.5 T. Several papers have also been published using prototype LF MRI scanners, typically 1.5 T scanners with magnetic inductance reduced to 0.55 T. However, these devices were not approved for clinical use and were used only in research mode. Therefore, we excluded these studies. Based on our analysis, we believe that there is potential for LF MRI machines, especially as a complement to the already established scanners using 1.5 and 3 T.

We believe that a study to identify suitable applications for low-field scanners would be essential. Lower costs for low-field equipment, as well as lower operating costs would make MRI examinations more affordable for patients and could also reduce waiting times for examinations. Early diagnosis can be important for further prevention of disease progression, especially in oncology.

With the increasing use of artificial intelligence in image processing, images from LF MRI machines could be sufficient for a growing number of diseases, and LF MRI machines could be used alone in hospitals without a 1.5 or 3 T scanner.

All identified studies [11,12] had an observational design without rigorous checking for the potential risk of bias, which could result in, for example, a higher likelihood of false negatives or failure to detect true differences in measurement results. The sample of patients in the identified studies was small (9 and 27) and not representative of the target population in terms of age, gender, heterogeneity of disease status, etc. Only one area was affected by the identified studies, namely brain imaging. In both identified studies, patients underwent repeated examinations using 0.55 T and 1.5 T MRI technologies within a short time interval. The results of the examinations were analysed by two raters (blinded and unblinded). The studies examined indicators of technical image quality (noise, contrast, etc.), lesion size, and ability to diagnose stroke. None of the identified studies investigated whether the use of a 0.55 T MRI device would lead to a change in patient health outcomes or clinical management compared with 1.5 T MRI scanners.

LF MR devices generally have less influence on the tissue under examination. For comparable examinations, LF MR devices have a lower SAR (specific absorption rate) at the patient and less heating in the area under examination, reducing the risk of interaction with implants and reducing acoustic noise. On the other hand, LF MR devices will produce images with a poorer signal-to-noise ratio (SNR) compared to conventional devices at similar examination times, resulting in lower geometric resolution, longer examination times, smaller field of view (FOV) and less benefit from the use of gadolinium contrast agent [13,14,15].

With new artificial intelligence-based image processing methods, the reconstructed methods can improve the signal-to-noise ratio, and the disadvantages of low magnetic fields can be suppressed. Also, artefacts caused by e.g., metal implants are lower in low magnetic field MRI systems compared to 1.5 T and 3 T. In general, lower magnetic induction leads to shorter T1 times and, conversely, longer T2 times, allowing shorter TR and longer spin echo acquisition sequences [13,14].

### 4.1. Limitations

The main limitation of this systematic review is the lack of evidence linking the results of imaging by different MRI technologies to the impact on clinically relevant outcomes, i.e., patient diagnosis, treatment, and clinical outcomes.

In the context of a systematic review, 39 studies were analysed after screening titles and abstracts. Only two studies met the predefined criteria. Excluded studies were assessed as inappropriate in terms of publication type [15,16,17,18,19,20,21,22,23,24] (e.g., review article) or study type [25,26,27] (e.g., commentary). Additional excluded studies compared the assessed technology with an inappropriate comparator [28,29,30,31,32,33,34,35,36,37,38,39,40] (e.g., Computed Tomography) or had an inappropriate study population [17] or outcome [43]. There were also studies excluded due to inappropriate technology [43,44,45,46,47,48,49,50,51]. These were comparative studies on prototypes that were not included because these prototypes represented experimental devices, were yet to be on the market, and had different technical parameters from devices on the market.

Also, only one type of scanner (Siemens) passed the original criteria, i.e., a whole-body scanner with a low inductance lower than 1 T that is approved for clinical usage.

The amount of literature on the diagnostic efficacy of MRI technologies may be limited in part due to the existing legislative framework for medical device regulation. Current, and especially previously applicable, regulations do not require device manufacturers to provide extensive clinical studies demonstrating clinical and diagnostic efficacy for the patient. For these reasons, manufacturers of MRI technologies are not “forced” to conduct similar studies that examine the impact of the technology on the clinical outcomes of patients.

### 4.2. Perspectives

LF MRI imaging systems generally require less space for installation and are lighter in weight than the conventional 1.5 T and 3 T MRI systems commonly used in clinical practice, making the installation requirements of LF MRI systems more flexible. The scanners are lighter in weight, whereas high-field MRI systems have a minimum weight of 3 tonnes. In addition, high-field systems require more separate rooms (examination rooms, workrooms and technical rooms with powerful electronics). Due to their ease of installation, LF MRI systems can be used in operating rooms, interventional theatres and emergency departments [1].

According to international studies, LF MR systems can offer cost-saving opportunities due to lower production costs and reduced installation and operation requirements. According to a study by Vosshenrich et al. [52], the acquisition cost of LF MR systems is approximately 43% lower than that of 1.5 T systems, comparable in software and coil equipment. Cost reductions also occur in shipping, as the weight of the instrument is 25% less than for the 1.5 T and 3 T systems, which can cut the cost per carrier in half. Weight and size also affect on-site transportation, which can sometimes be much simpler (without a crane, without building reconstruction and with other modifications). In addition, according to the authors of this study, there is no need for refrigeration equipment due to the minimal amount of helium. Significant savings also result from lower requirements for ventilation, cooling, electromagnetic shielding and wiring. According to the authors, MRI devices allow for savings in installation and maintenance costs compared to higher-intensity MRI systems. Lower low-field equipment and operating costs could also lead to improved patient care in developing countries and better access to MRI scans.

## 5. Conclusions

Technical development has contributed significantly to the improvement of the image quality of LF MRI systems and their diagnostic efficacy. The low magnetic induction value means that in specific cases, the LF MRI system is preferable to standard clinical devices with 1.5 T and 3 T. However, it has some disadvantages [8]. Therefore, low magnetic field induction devices cannot currently be seen as fully-fledged devices suitable for comprehensive examinations of a wide range of patients in many different areas, but are rather suitable as a complement to standard 1.5 T and 3 T magnetic induction devices, but this may change with further development, particularly in the field of SW [53].

According to a literature search, there is limited peer-reviewed scientific evidence on the accuracy and diagnostic efficacy of LF MRI compared to 1.5 T devices. Well-designed, larger clinical trials are needed to draw valid conclusions regarding how effective and accurate the new 0.55 T MRI technology is compared to conventional 1.5 T MRI technology.

LF MRI systems can be used especially in specific examinations where low magnetic field induction seems to be advantageous, such as reduction of artefacts from metal implants or reduction of the influence of different susceptibility of the examined tissues, e.g., lung tissue [15]. Due to the lower SAR, this technique is also suitable where it is necessary to limit possible interaction with medical devices or effects on metal implants [13,52].

Given the anticipated further development, it can be expected that LF MRI systems will be a suitable complement to existing MRI imaging instrumentation, reducing the cost of MRI examinations and “lightening” the burden on standard clinical devices [8].

## Figures and Tables

**Table 1:** Excluded studies.

Author of the Study	About the Study	Reason for Exclusion
Allam 2018 [12]	This study is focused on the evaluation of LF-MRI cryptorchidism. High specificity and diagnostic accuracy were found.	Unsuitable technology: 0.4 T Aperto MRI Hitachi—open
Campbell-Washburn 2019 [13]	The study examines lung imaging using a modified MRI system to 0.55 T. According to the results of LF-MRI, the visualisation of the lung parenchyma is improved.	Unsuitable type of publication: Proposal
Montesino 2019 [14]	The study evaluated rheumatologic diseases by LF-MRI. LF-MRI may be helpful as a complementary examination.	Unsuitable type of publication: Conference paper
Anisimov 2020 [15]	The study focused on imaging of the brain using 0.5 T research MRI using the macromolecular proton fraction (MPF) mapping method. The results demonstrate that MPF is a valuable absolute scale for measuring myelin content using MRI at various magnetic field strengths.	Unsuitable technology:MRI for research purposes Tomikon S50
Schröder 2020 [16]	The study focused on total knee arthroplasty patients. Low-field MRI shows similar diagnostic value to CT and could be a cost-effective alternative without radiation.	Unsuitable technology: 0.25 T Esaote G-scan Brio—open
Basar 2021 [17]	The study focused on the evaluation of artefacts. Steel is a source of artefacts independent of the MRI field; an MRI indication <0.55 T may minimise these artefacts.	Unsuitable population: Evaluation of artefact
Harper 2021 [18]	Image quality for hydrocephalus treatment planning was evaluated. Images with lower quality than is customarily acceptable can be helpful for hydrocephalus treatment planning.	Unsuitable technology: Prototype
Campbell-Washburn 2021 [19]	A 3D FISP MRF sequence was implemented for the 0.55 T system. The feasibility of whole-brain T1 and T2 mapping using MRF in healthy volunteers was demonstrated.	Unsuitable comparator: Phantom MRI
Wang 2021 [20]	The study focused on LF-MRI brain imaging, and sensitivity was assessed. It showed that the blood oxygenation level-dependent contrast is sufficiently high and can be used to extend the range of LFMRI applications.	Unsuitable comparator: was no comparator
Bhattacharya 2021 [21]	Using high-performance 0.55 T MRI, experts were able to perform simultaneous imaging of pulmonary structure and regional function in patients with lymphangioleiomyomatosis.	Unsuitable comparator: Prototype
Javed 2021 [22]	An MRI scan of the lungs was performed. Fast image reconstruction was implemented for diagnostic quality, which was validated and evaluated in our technique in healthy volunteers, patients with lung nodules, and patients with COVID-19 infection.	Unsuitable comparator: Prototype
Campbell-Washburn 2021 [23]	An MRI scan of the lungs was performed and compared with CT scans. The MRI scan was of sufficient quality to detect pathologies	Unsuitable comparator: Computed Tomography
Campbell-Washburn 2021 [24]	This publication deals with LF-MRI of the lungs in a patient with COVID-19 and focuses on a case report.	Unsuitable type of publication: Reviews and commentary
Heiss 2021 [25]	This is a case study of a patient with COVID-19, for which LFMRI was used. LF-MRI enables the precise visualisation of persistent pulmonary changes, which are consistent with CT performed on the same day.	Unsuitable type of publication: Case report
Norris 2021 [26]	This publication focuses on expert commentary on the issues surrounding conventional MRI and LFMRI.	Unsuitable type of study: Commentary
Chiragzada 2022 [27]	This study is focused on targeted biopsy using MRI-guided biopsy over transrectal ultrasound. The targeted MRI approach significantly benefited the patient by favourably impacting the care.	Unsuitable technology: Transperineal biopsy
Porrelli 2022 [28]	The present study demonstrates the synergistic use of LF-NMR and micro-CT in detecting structural morphometric changes in the trabecular bone of osteoporosis specimens compared to osteoarthritis.	Unsuitable technology: Spectrometer—Bruker Mini spec mq 20
Qiu 2022 [29]	A comparison of susceptibility-weighted imaging (SWI) in 0.5 T and 1.5 T was carried out, demonstrating the capability to identify magnetic susceptibility differences between variable tissues, especially the blood veins.	Unsuitable technology: Prototype
Stamenkovic 2022 [30]	The study focuses on LF-MRI joint examinations. The findings suggest that MRI should be used in symptomatic patients.	Unsuitable technology: 0.2 T Artroscan—open
Bhattacharya 2022 [31]	This study focused on the LF-MRI imaging of lungs with simultaneous imaging of pulmonary structure and regional function in patients with lymphangioleiomyomatosis.	Unsuitable comparator: Computed tomography
Lévy 2022 [32]	Functional pulmonary examinations using free-breathing LF-MRI revealed potential quantitative markers of impaired lung function in patients with persistent symptoms after COVID-19 infection, potentially complementing morphologic imaging.	Unsuitable comparator: Computed tomography
Seemann 2022 [33]	The study focuses on imaging water in the lungs using cardiovascular magnetic resonance. The results suggest that LF-MRI helps assess changes in the lungs.	Unsuitable comparator: Phantom MRI
Azour 2022 [34]	The study evaluated the detection of lung by LF-MRI versus clinically-acquired chest CT images in a cohort of post-COVID patients. LF MRI 0.55 T demonstrates fair-to-moderate inter-reader concordance.	Unsuitable comparator: Computed tomography
Wujciak 2022 [35]	Measurement parameters and examination protocols were analysed to see if they met the requirements. The conclusions confirm that LF-MRI can partially replace conventional MRI.	Unsuitable comparator: Compared different scenarios
Anzai 2022 [36]	This is an expert commentary on the issues of conventional MRI and LF-MRI.	Unsuitable type of publication: Reviews and commentary
Breit 2022 [37]	This is an expert commentary on the issues of conventional MRI and LF-MRI.	Unsuitable type of publication: Reviews and commentary
Cawley 2022 [38]	This is an editorial focused on new possibilities offered by low-field technology.	Unsuitable type of publication: Editorial
Sekhon 2022 [39]	Editorial about problematic MRI—view into hypoxic-ischemic brain injury after cardiac arrest	Unsuitable type of publication: Editorial
Arnold 2022 [40]	The study focused on imaging multiple sclerosis (MS). The authors found that a porTable 64 mT scanner was sensitive in MS patients and that an automated algorithm designed for 3 T image segmentation could be applied.	Unsuitable type of publication: 64 mT MRI vs. 3 T MRI
Breit 2022 [41]	This is a review focused on the effects, image acquisition, and diagnostic quality of the examination.	Unsuitable type of study: Review
Mertz 2022 [42]	Abstract about New Efforts in Biomedical Imaging.	Unsuitable type of study: Abstract
Rusche 2022 [43]	The study was to assess patient comfort when imaged on a newly introduced 0.55 T low-field magnetic resonance (MR) scanner system with a broader bore opening compared to a conventional 1.5 T MR scanner system.	Unsuitable output: Evaluated patient comfort
Klippel 2023 [44]	The study focuses on a case report of a neurological patient	Unsuitable technology: 0.4 T Fuji Aperto Lucent Plus- open
Tian 2023 [45]	Contemporary LF-MR scanners equipped with high-performance gradient systems allow the use of contrast-optimal flip-angles for multi-slice accelerated examinations without compromising image quality	Unsuitable comparator: Was no comparator
Li 2023 [46]	The study focuses on LF-MRI lung imaging. According to the literature, the results are similar to a 1.5 T MRI in the evaluation of lung parenchyma.	Unsuitable comparator: Was no comparator
Heiss 2023 [47]	LF-MRI evaluated lung parenchyma in children and adolescents post-COVID-19 compared to healthy controls. LF-MRI showed persistent pulmonary dysfunction in children and adolescents.	Unsuitable comparator: Clinical symptoms and serologic parameter
Paltiel 2023 [48]	This is an expert commentary on the issues of LF-MRI.	Unsuitable type of publication: Review and commentary

**Table 2:** Basic characteristics of the studies included in the analysis.

Study	Study Design	Number of Patients	Population	Area
Osmanodja 2023 [49]	Cross-over, single-centre, retro-prospective	9 patients	Patients with suspected intracranial aneurysm	Brain
Rusche 2022 [50]	Cross-over and case-control, single-centre, prospective	A total of 27 patients were included in the study (17 in the stroke group and 10 in the control group)	Patients with suspected stroke or transient ischemic attack	Brain

**Table 3:** Results of comparison of diagnostic efficacy of two technologies.

Study	Outputs	Measurements	Results
Osmanodja 2023 [49]	Size of aneurysm	TOF MRA Two unblinded raters	Insignificant differences in aneurysm size
Rusche 2022 [50]	10-point scale (overall image quality, resolution, noise, contrast, diagnostic quality) Reading study (stroke yes/no; numbers/locations of DWI and FLAIR lesions) Image segmentation— lesion size	DWI/ADC and FLAIR sequences Two unblinded neuroradiologists Two blinded neuroradiologists Two radiologists	DWI/ADC: overall image quality, resolution, contrast, and diagnostic quality were significantly better in the 1.5 T MRI device, and image noise assessment was significantly better in the 0.55 T MRI device than in the 1.5 T MRI device. FLAIR: overall image quality, noise, and diagnostic quality were significantly better with 1.5 T MRI technology, and there were no significant differences between technologies for resolution and contrast. DWI/ADC: rater 1—same sensitivity and specificity values (92.9% and 100%), rater 2—sensitivity 85.7% vs. 100%, same specificity 100% No significant differences between technologies

## Data Availability

Data sharing is not applicable.

## Supplementary Materials

The following supporting information can be downloaded at: https://www.mdpi.com/article/10.3390/diagnostics14141564/s1, Table S1: Search strategy. Table S2: QUADAS-2. Table S3: Quality assessment of the studies included in the systematic review. Table S2 File S1: PRISMA Checklist. File S2: Studies excluded in the systematic review.
