# Supplementary material for: Assessment of the Diagnostic Efficacy of Low-Field Magnetic Resonance Imaging: A Systematic Review
Source: Diagnostics (Basel). 2024 Jul 19;14(14):1564. doi: 10.3390/diagnostics14141564 (PMC11276230; doi:10.3390/diagnostics14141564)
Supplement: Supplementary file 1 [file diagnostics-14-01564-s001.zip › Studies excluded in the systematic review.pdf]

Studies excluded in the systematic review

Unsuitable type of publicaton

1 ANZAI, Yoshimi a MOY, Linda. Point-of-Care Low-Field-Strength MRI Is Moving Beyond the Hype. *Radiology*. 2022, **305**(3), 672-673. Dostupné z: 10.1148/radiol.221278

2 BREIT, Hanns-Christian a BAUMAN, Grzegorz. Morphologic and Functional Assessment of Sarcoidosis Using Low-Field MRI. *Radiology*. 2022, **303**(2), 255-255. Dostupné z: 10.1148/radiol.211760

3 CAMPBELL-WASHBURN, Adrienne E. 2019 American Thoracic Society BEAR Cage Winning Proposal: Lung Imaging Using High-Performance Low-Field Magnetic Resonance Imaging. *American journal of respiratory and critical care medicine*. 2020, **201**(11), 1333-1336. Dostupné z: 10.1164/rccm.201912-2505ED

4 CAMPBELL-WASHBURN, Adrienne E., SUFFREDINI, Anthony F. a CHEN, Marcus Y. High-Performance 0,55-T Lung MRI in Patient with COVID-19 Infection. *Radiology*. 2021, **299**(2), E246-E247. Dostupné z: 10.1148/radiol.2021204155

5 CAWLEY, Paul A., NOSARTI, Chiara a EDWARDS, A. David. In-unit neonatal magnetic resonance imaging-new possibilities offered by low-field technology. *Journal of perinatology : official journal of the California Perinatal Association*. 2022, **42**(7), 843-844. Dostupné z: 10.1038/s41372-022-01401-w

6 HEISS, Rafael, GRODZKI, David M., HORGER, Wilhelm, UDER, Michaela kol. High-performance low field MRI enables visualization of persistent pulmonary damage after COVID-19. *Magnetic resonance imaging*. 2021, **76**, 49-51. Dostupné z: 10.1016/j.mri.2020.11.004

7 OKAR, S. V., NAIR, G., KAWATRA, K. D., BY, S.a kol. Sensitivity of ultra-low-field magnetic resonance imaging for white matter lesions and leptomeningeal enhancement in multiple sclerosis. *MULTIPLE SCLEROSIS JOURNAL*. 2021, **27**(2_SUPPL), 414-415. Dostupné z,

8 PALTIEL, Harriet J. Low-field MRI and ventilation-perfusion mismatch after pediatric COVID-19. *Radiology*. 2022, 222360-222360. Dostupné z: 10.1148/radiol.222360

9 RUIZ MONTESINO, Maria Dolores a MENDOZA MENDOZA, Dolores. Carpal tunnel syndrome due to a tophus: Low-field magnetic resonance image. *Reumatologia clinica*. 2019, **15**(6), e149-e150. Dostupné z: 10.1016/j.reuma.2017.07.021

10 SEKHON, Mypinder S. a GRIESDALE, Donald E. Low field magnetic resonance imaging: A "beds-eye-d" view into hypoxic ischemic brain injury after cardiac arrest. *Resuscitation*. 2022, **176**, 55-57. Dostupné z: 10.1016/j.resuscitation.2022.05.010

Unsuitable technology

1 ALLAM, Mfab, ELIAN, M. M. M., RAHMAN, A. M. A. a ALLAM, Fafab. The utility of chemical shift imaging and related fat suppression as standalone technique in cryptorchidism using low field MRI. *EGYPTIAN JOURNAL OF RADIOLOGY AND NUCLEAR MEDICINE*. 2018, **49**(4), 1140-1144. Dostupné z: 10.1016/j.ejrnm.2018.07.008

2 ANISIMOV, Nikolay V., PAVLOVA, Olga S., PIROGOV, Yury A. a YARNYKH, Vasily L. Three-dimensional fast single-point macromolecular proton fraction mapping of the human brain at 0.5 Tesla. *Quantitative imaging in medicine and surgery*. 2020, **10**(7), 1441-1449. Dostupné z: 10.21037/qims-19-1057

3 HARPER, Joshua R., CHERUKURI, Venkateswararao, O'REILLY, Tom, YU, Mingzhaoa kol. Assessing the utility of low resolution brain imaging: treatment of infant hydrocephalus. *NeuroImage. Clinical*. 2021, **32**, 102896-102896. Dostupné z: 10.1016/j.nicl.2021.102896

4 CHIRAGZADA, Selin, SATYA, Poorvi, MACALUSO, Joseph N., Jr., VENKATARAMAN, Srirama S.a kol. Advantageous Detection of Significant Prostate Cancer Using a Low-Field, Office-Based MRI System. *Cureus*. 2022, **14**(12), e32105-e32105. Dostupné z: 10.7759/cureus.32105

5 KLIPPEL, Ekkehard a MOSHAGEN, Volker. [Neurological manifestation of cerebrotendinous xanthomatosis-Clinical findings and cranial imaging in low-field MRI]. *Der Nervenarzt*. 2022. Dostupné z: 10.1007/s00115-022-01402-2

6 PORRELLI, Davide, ABRAMI, Michela, PELIZZO, Patrizia, FORMENTIN, Cristinaa kol. Trabecular bone porosity and pore size distribution in osteoporotic patients - A low field nuclear magnetic resonance and microcomputed tomography investigation. *Journal of the mechanical behavior of biomedical materials*. 2022, **125**, 104933-104933. Dostupné z: 10.1016/j.jmbbm.2021.104933

7 QIU, Yueqi, BAI, Haoran, CHEN, Hao, ZHAO, Yuea kol. Susceptibility-weighted imaging at high-performance 0.5T magnetic resonance imaging system: Protocol considerations and experimental results. *Frontiers in neuroscience*. 2022, **16**, 999240-999240. Dostupné z: 10.3389/fnins.2022.999240

8 SCHRODER, Femke F., POST, Corine E., VAN RAAK, Sjoerd M., SIMONIS, Frank F. J.a kol. The diagnostic potential of low-field MRI in problematic total knee arthroplasties - a feasibility study.

Journal of experimental orthopaedics. 2020, **7**(1), 59-59. Dostupné z: 10.1186/s40634-020-00274-2

9 STAMENKOVIC, B., STOJANOVIC, S., ZIVKOVIC, V., DJORDJEVIC, D.a kol. Low-Frequency Magnetic Resonance Imaging Identifies Hand Joint Subclinical Inflammation in Systemic Sclerosis. *DIAGNOSTICS*. 2022, **12**(9). Dostupné z: 10.3390/diagnostics12092165

Unsuitable comparator

1 AZOUR, Lea, CONDOS, Rany, KEERTHIVASAN, Mahesh B., BRUNO, Marya kol. Low-field 0,55T MRI for assessment of pulmonary groundglass and fibrosis-like opacities: Inter-reader and inter-modality concordance. *European journal of radiology*. 2022, **156**, 110515-110515. Dostupné z: 10.1016/j.ejrad.2022.110515

2 BHATTACHARYA, Ipshita, RAMASAWMY, Rajiv, JAVED, Ahsan, CHEN, Marcus Y.a kol. Oxygen-enhanced functional lung imaging using a contemporary 0,55T MRI system. *NMR in biomedicine*. 2021, **34**(8), e4562-e4562. Dostupné z: 10.1002/nbm.4562

3 BHATTACHARYA, Ipshita, RAMASAWMY, Rajiv, JAVED, Ahsan, LOWERY, Margareta kol. Assessment of Lung Structure and Regional Function Using 0,55 T MRI in Patients With Lymphangioleiomyomatosis. *Investigative radiology*. 2022, **57**(3), 178-186. Dostupné z: 10.1097/RLI.0000000000000832

4 CAMPBELL-WASHBURN, Adrienne E., JIANG, Yun, KORZDORFER, Gregor, NITTKA, Mathiasa kol. Feasibility of MR fingerprinting using a high-performance 0,55T MRI system. *Magnetic resonance imaging*. 2021, **81**, 88-93. Dostupné z: 10.1016/j.mri.2021.06.002

5 CAMPBELL-WASHBURN, Adrienne E., MALAYERI, Ashkan A., JONES, Elizabeth C., MOSS, Joela kol. T2-weighted Lung Imaging Using a 0,55-T MRI System. *Radiology. Cardiothoracic imaging*. 2021, **3**(3), e200611-e200611. Dostupné z: 10.1148/ryct.2021200611

6 HEISS, Rafael, TAN, Lina, SCHMIDT, Sandy, REGENSBURGER, Adrian P.a kol. Pulmonary Dysfunction after Pediatric COVID-19. *Radiology*. 2022, 221250-221250. Dostupné z: 10.1148/radiol.221250

7 JAVED, Ahsan, RAMASAWMY, Rajiv, O'BRIEN, Kendall, MANCINI, Christinea kol. Self-gated 3D stack-of-spirals UTE pulmonary imaging at0,55T. *Magnetic resonance in medicine*. 2022, **87**(4), 1784-1798. Dostupné z: 10.1002/mrm.29079

8 LEVY, Simon, HEISS, Rafael, GRIMM, Robert, GRODZKI, Davida kol. Free-Breathing Low-Field MRI of the Lungs Detects Functional Alterations Associated With Persistent Symptoms After COVID-19

Infection. *Investigative radiology*. 2022, **57**(11), 742-751. Dostupné z: 10.1097/RLI.0000000000000892

9 LI, Bochao, LEE, Nam G., CUI, Sophia X. a NAYAK, Krishna S. Lung parenchyma transverse relaxation rates at 0,55 T. *Magnetic resonance in medicine*. 2022. Dostupné z: 10.1002/mrm.29541

10 SEEMANN, Felicia, JAVED, Ahsan, CHAE, Rachel, RAMASAWMY, Rajiva kol. Imaging gravity-induced lung water redistribution with automated inline processing at 0,55 T cardiovascular magnetic resonance. *Journal of cardiovascular magnetic resonance : official journal of the Society for Cardiovascular Magnetic Resonance*. 2022, **24**(1), 35-35. Dostupné z: 10.1186/s12968-022-00862-4

11 TIAN, Ye, CUI, Sophia X., LIM, Yongwan, LEE, Nam G.a kol. Contrast-optimal simultaneous multi-slice bSSFP cine cardiac imaging at 0,55T. *Magnetic resonance in medicine*. 2023, **89**(2), 746-755. Dostupné z: 10.1002/mrm.29472

12 WANG, Yicun, VAN GELDEREN, Peter, DE ZWART, Jacco A., CAMPBELL-WASHBURN, Adrienne E.a kol. FMRI based on transition-band balanced SSFP in comparison with EPI on a high-performance 0,55 T scanner. *Magnetic resonance in medicine*. 2021, **85**(6), 3196-3210. Dostupné z: 10.1002/mrm.28657

13 WUJCIAK, Detlef. [Modern mid-field magnetic resonance imaging in private practice : Field report]. *Der Radiologe*. 2022, **62**(5), 405-409. Dostupné z: 10.1007/s00117-022-00988-7

Unsuitable population

1 BASAR, Burcu, SONMEZ, Merdim, YILDIRIM, Dursun Korel, PAUL, Rama kol. Susceptibility artifacts from metallic markers and cardiac catheterization devices on a high-performance 0,55T MRI system. *Magnetic resonance imaging*. 2021, **77**, 14-20. Dostupné z: 10.1016/j.mri.2020.12.002

Unsuitable type of study

1 BREIT, Hanns-Christian, VOSSHENRICH, Jan, BACH, Michael a MERKLE, Elmar M. [New clinical applications for low-field magnetic resonance imaging : Technical and physical aspects]. *Der Radiologe*. 2022, **62**(5), 394-399. Dostupné z: 10.1007/s00117-022-00967-y

2 MERTZ, L. New Efforts in Biomedical Imaging. *IEEE Pulse*. 2022, **13**(4), 2-7. Dostupné z: 10.1109/MPULS.2022.3191382

3 NORRIS, David Gordon a WEBB, Andrew. This house proposes that low field and high field MRI are by destiny worst enemies, and can never be the best of friends! *Magma (New York, N.Y.)*. 2021, **34**(4), 475-477. Dostupné z: 10.1007/s10334-021-00940-1

Unsuitable output

1 RUSCHE, Thilo, VOSSHENRICH, Jan, WINKEL, David J., DONNERS, Ricardoa kol. More Space, Less Noise-New-generation Low-Field Magnetic Resonance Imaging Systems Can Improve Patient Comfort: A Prospective 0,55T-1,5T-Scanner Comparison. *Journal of clinical medicine*. 2022, **11**(22). Dostupné z: 10.33
