# Supplementary material for: Assessment of the Diagnostic Efficacy of Low-Field Magnetic Resonance Imaging: A Systematic Review
Source: Diagnostics (Basel). 2024 Jul 19;14(14):1564. doi: 10.3390/diagnostics14141564 (PMC11276230; doi:10.3390/diagnostics14141564)
Supplement: Supplementary file 1 [file diagnostics-14-01564-s001.zip › Tables.pdf]

Table S1: **Search strategy**

MEDLINE

	Search Query
1	TS = ("0,55T" OR "0,55 T" OR "0,55-T" OR ".55T" OR ".55 T" OR ".55-T" OR "0,55 Tesla" OR ".55 Tesla")
2	TS = ("Low-field" OR "Low field" OR "Low fielded" OR "Mid-field" OR "Mid field" OR "Mid fielded")
3	TS = ("Free.Max" OR "Free MAX" OR Free.Star OR "FREE STAR")
4	#1 OR #2 OR #3
5	TS = (MRI OR "magnetic resonance imag*" OR "MR imag*" OR "MR scan*" OR “MR tomograph*”)
6	MHX = (magnetic resonance imaging)
7	#5 OR #6
8	#4 AND #7
9	MHX=(Animals OR Animal Experimentation OR "Models, Animal" OR Vertebrates) NOT MHX=(Humans OR Human experimentation)
10	#8 NOT #9
11	#10 AND PY = (2018-2024)

PubMed

The search studies were limited to records not indexed in the MEDLINE database.

	Search Query
1	"0,55T"[Title/Abstract] OR "0,55 T"[Title/Abstract] OR "0,55-T"[Title/Abstract] OR ".55T"[Title/Abstract] OR ".55 T"[Title/Abstract] OR ".55-T"[Title/Abstract] OR "0,55 Tesla"[Title/Abstract] OR ".55 Tesla"[Title/Abstract]
2	"Low-field"[Title/Abstract] OR "Low field"[Title/Abstract] OR "Low fielded"[Title/Abstract] OR "Mid-field"[Title/Abstract] OR "Mid field"[Title/Abstract] OR "Mid fielded"[Title/Abstract]
3	"Free.Max"[Title/Abstract] OR "Free MAX"[Title/Abstract] OR Free.Star[Title/Abstract] OR "FREE STAR"[Title/Abstract]
4	#1 OR #2 OR #3
5	MRI[Title/Abstract] OR "magnetic resonance imag*"[Title/Abstract] OR "MR imag*"[Title/Abstract] OR "MR scan*"[Title/Abstract] OR "MR tomograph*"[Title/Abstract]
6	magnetic resonance imaging[MeSH Terms]
7	#5 OR #6
8	#4 AND #7
9	"Animals"[MeSH Terms] OR "Animal Experimentation"[MeSH Terms] OR "Models, Animal"[MeSH Terms] OR "Vertebrates"[MeSH Terms]

10	"Humans"[MeSH Terms] OR "Human experimentation"[MeSH Terms]
11	#9 NOT #10
12	#8 NOT #11
13	pubstatusaheadofprint OR publisher[sb] OR pubmednotmedline[sb]
14	#12 AND #13
15	("2018/01/01"[Date - Publication] : "3000"[Date - Publication])
16	#14 AND #15

Web of science

	Search Query
1	TS = ("0,55T" OR "0,55 T" OR "0,55-T" OR ".55T" OR ".55 T" OR ".55-T" OR "0,55 Tesla" OR ".55 Tesla")
2	TS = ("Low-field" OR "Low field" OR "Low fielded" OR "Mid-field" OR "Mid field" OR "Mid fielded")
3	TS = ("Free.Max" OR "Free MAX" OR Free.Star OR "FREE STAR")
4	#1 OR #2 OR #3
5	TS = (MRI OR "magnetic resonance imag*" OR "MR imag*" OR "MR scan*" OR “MR tomograph*”)
6	#4 AND #5
7	#6 AND PY = (2018-2024)
8	TS=(sensitiv* OR detect* OR accura* OR specific* OR reliab* OR positive OR negative OR diagnos*)
9	#7 AND #8

Scopus

(TITLE-ABS-KEY (0,55t OR {0,55 T} OR {0,55-T} OR .55t OR {.55 T} OR {.55-T} OR {0,55 Tesla} OR {.55 Tesla}) OR TITLE-ABS-KEY ({Low-field} OR {Low field} OR {Low fielded} OR {Mid-field} OR {Mid field} OR {Medium field} OR {Mid fielded}) OR TITLE-ABS-KEY (free.max OR {Free MAX} OR free.star OR {FREE STAR})) AND TITLE-ABS-KEY (mri OR "magnetic resonance imaging" OR "MR imag*" OR "MR scan*") AND TITLE-ABS-KEY (sensitiv* OR detect* OR accura* OR specific* OR reliab* OR positive OR negative OR diagnos*) AND PUBYEAR > 2018 AND PUBYEAR < 2024

CENTRAL

ID	Search
#1	(0,55T OR "0,55 T" OR "0,55-T" OR .55T OR ".55 T" OR ".55-T" OR "0,55 Tesla" OR ".55 Tesla"):ti,ab,kw
#2	("Low-field" OR "Low field" OR "Low fielded" OR "Mid-field" OR "Mid field" OR "Medium field" OR "Mid fielded"):ti,ab,kw
#3	("Free.Max" OR "Free MAX" OR Free.Star OR "FREE STAR"):ti,ab,kw
#4	#1 OR #2 OR #3
#5	(MRI OR "magnetic resonance imag*" OR "MR imag*" OR "MR scan*"):ti,ab,kw
#6	MeSH descriptor: [Magnetic Resonance Imaging] explode all trees
#7	#4 AND #6

#8	#7 with Cochrane Library publication date from Jan 2018 to present

Table S2: **QUADAS-2**

Phase 3: Risk of bias and applicability judgments

QUADAS-2 is structured so that 4 key domains are each rated in terms of the risk of bias and the concern regarding applicability to the research question (as defined above). Each key domain has a set of signalling questions to help reach the judgments regarding bias and applicability.

DOMAIN 1: PATIENT SELECTION

A. Risk of Bias

Describe methods of patient selection:

- ❖ Was a consecutive or random sample of patients enrolled? Yes/No/Unclear
- ❖ Was a case-control design avoided? Yes/No/Unclear
- ❖ Did the study avoid inappropriate exclusions? Yes/No/Unclear

Could the selection of patients have introduced bias? RISK: LOW/HIGH/UNCLEAR

B. Concerns regarding applicability

Describe included patients (prior testing, presentation, intended use of index test and setting):

Is there concern that the included patients do not match the review question? CONCERN: LOW/HIGH/UNCLEAR

DOMAIN 2: INDEX TEST(S)

If more than one index test was used, please complete for each test.

A. Risk of Bias

Describe the index test and how it was conducted and interpreted:

- ❖ Were the index test results interpreted without knowledge of the results of the reference standard? Yes/No/Unclear
- ❖ If a threshold was used, was it pre-specified? Yes/No/Unclear

Could the conduct or interpretation of the index test have introduced bias? RISK: LOW /HIGH/UNCLEAR

B. Concerns regarding applicability

Is there concern that the index test, its conduct, or interpretation differ from the review question? CONCERN: LOW /HIGH/UNCLEAR

DOMAIN 3: REFERENCE STANDARD

A. Risk of Bias

Describe the reference standard and how it was conducted and interpreted:

- ❖ Is the reference standard likely to correctly classify the target condition? Yes/No/Unclear
- ❖ Were the reference standard results interpreted without knowledge of the results of the index test? Yes/No/Unclear

Could the reference standard, its conduct, or its interpretation have introduced bias? **RISK: LOW /HIGH/UNCLEAR**

B. Concerns regarding applicability

Is there concern that the target condition as defined by the reference standard does not match the review question? **CONCERN: LOW /HIGH/UNCLEAR**

DOMAIN 4: FLOW AND TIMING

A. Risk of Bias

Describe any patients who did not receive the index test(s) and/or reference standard or who were excluded from the 2x2 table (refer to flow diagram):

Describe the time interval and any interventions between index test(s) and reference standard:

- ❖ Was there an appropriate interval between index test(s) and reference standard? Yes/No/Unclear
- ❖ Did all patients receive a reference standard? Yes/No/Unclear
- ❖ Did patients receive the same reference standard? Yes/No/Unclear
- ❖ Were all patients included in the analysis? Yes/No/Unclear

Could the patient flow have introduced bias? **RISK: LOW /HIGH/UNCLEAR**

Table S3: **Quality assessment of the studies included in the systematic review**

**Study**	**Risk of Bias**				**Applicability Concerns**		
	Patient Selection	Index Test	Reference Standard	Flow and Timing	Patient Selection	Index Test	Reference Standard
**Osmanodja 2023**	Low	Low	Low	High	Low	Low	Low
**Rusche 2022**	High	Low	Low	Low	Low	Low	Low
